# Characteristics of HPV integration in cervical adenocarcinoma and squamous carcinoma

**DOI:** 10.1007/s00432-023-05494-4

**Published:** 2023-11-15

**Authors:** Yuxin Bi, Junbo Hu, Ling Zeng, Gang Chen, Hongning Cai, Huang Cao, Quanfu Ma, Xufeng Wu

**Affiliations:** 1 Maternal and Child Health Hospital of Hubei Province, Tongji Medical College, Huazhong University of Science and Technology, Wuhan, China; 2 Hubei Clinical Medical Research Center for Gynecologic Malignancy, Wuhan, China; 3 Department of Obstetrics and Gynecology, Tongji Hospital, Tongji Medical College, Huazhong University of Science and Technology, Wuhan, Hubei China; 4 Department of Pathology, Maternal and Child Health Hospital of Hubei Province, Wuhan, China; 5 Hubei Provincial Center for Medical Genetics, Wuhan, China

**Keywords:** HPV integration, HPV capture sequencing, Cervical adenocarcinoma, Cervical squamous carcinoma, Characterization of integration, Carcinogenesis of HPV integration

## Abstract

**Purpose:**

HPV integration usually occurs in HPV-related cancer, and is the main cause of cancer. But the carcinogenic mechanism of HPV integration is unclear. The study aims to provide a theoretical basis for understanding the pathogenesis of cervical adenocarcinoma (AC) and cervical squamous carcinoma (SCC).

**Methods:**

We used HPV capture sequencing to obtain HPV integration sites in AC and SCC, and analyzed cytobands, distribution of genetic and genomic elements, identified integration hotspot genes, clinicopathological parameters, breakpoints of HPV16 and performed pathway analysis. Then we conducted immunohistochemical (IHC) assay to preliminarily verify the expression of most frequently integrated genes in AC, STARD3 and ERBB2.

**Results:**

The results revealed that the most frequently observed integrated cytoband was 17q12 in AC and 21p11.2 in SCC, respectively. The breakpoints in both AC and SCC were more tended to occur within gene regions, compared to intergenetic regions. Compared to SCC samples, AC samples had a higher prevalence of genomic elements. In AC, HPV integration has no significantly difference with clinicopathological parameters, but in SCC integration correlated with differentiation (*P* < 0.05). Breakpoints of HPV in SCC located in LCR more frequently compared to AC, which destroyed the activation of promoter p97. Hotspot genes of HPV integration were STARD3 and ERBB2 in AC, and RNA45S rDNA and MIR3648-1 in SCC, respectively. Meanwhile, we preliminarily proved that the expression of STARD3 and ERBB2, the most frequently integrated genes, would increase after integration.

**Conclusion:**

These results suggested that HPV may utilize the powerful hosts’ promoters to express viral oncogenes and overexpression of viral oncogenes plays a significant role in the carcinogenesis of SCC. In AC, HPV integration may affect hosts’ oncogenes, and the dysregulation of oncogenes may primarily contribute to progression of AC.

**Supplementary Information:**

The online version contains supplementary material available at 10.1007/s00432-023-05494-4.

## Introduction

Cervical cancer is a common gynecological tumor (Li et al. 2019), with approximately 604,000 new cases and 342,000 deaths reported worldwide in 2020 (Sung et al. 2021). Squamous carcinoma (SCC) and adenocarcinoma (AC) are the two main pathological types of cervical cancer. With widespread cervical cancer screening, the mortality of SCC has significantly decreased. However, the mortality of AC remains high because of low detection sensitivity, young age of onset, low survival rate, and high rate of metastasis (Dong Hong et al. 2010; Irie et al. 2000; Shimada et al. 2006).

To our knowledge, persistence of HPV infection is the main cause of cervical cancer. HPV is a small nonenveloped double-stranded DNA virus (Hoppe-Seyler et al. 2018) that establishes its genome as episomes in proliferating basal cells (Bedell et al. 1991). The expression of viral oncoproteins E6 and E7 promotes the progression of cervical cancer. Although HPV integration is critical, the carcinogenic mechanism of integration is unclear. Studies have reported that HPV integration sites tend to be located in repetitive regions of the host genome, with some sites near cancer-relevant genes (Li et al. 2013), and identified integration hotspot genes, including POU5F1B, FHIT, and KLF12 (Hu et al. 2015). Kamal et al. studied the pattern of HPV integration in SCC and AC and reported that HPV integration signatures are not associated with histological subtypes (Kamal et al. 2021). Because the number of AC cases is limited, most studies on HPV integration have focused on SCC (Cancer Genome Atlas Research et al. 2017; Hu et al. 2015; Ojesina et al. 2014; Li et al. 2013); however, none have evaluated the discrepancy in integration features between SCC and AC. We systematically and comprehensively studied the differences of HPV integration characteristics between SCC and AC. The aim of this study is to understand HPV integration-mediated carcinogenesis in AC and SCC, and to provide a useful information for researches on treatment and screen of cervical cancer.

## Materials and methods

### Sample collection

A total of 66 AC samples were collected from the Maternal and Child Health Hospital of Hubei Province between 2015 and 2020. The clinicopathological parameters included age, HPV type, FIGO stage, differentiation, and lymph node metastasis.

### HPV capture sequencing

HPV capture sequencing was employed to identify breakpoints of the virus and host. The detailed method was as described in a previous study (Hu et al. 2023). Integrated sites with reads ≥ 7 were deemed valid integration sites. The sequencing data for 95 cases of SCC, sequenced using the same method, were provided by Tongji Hospital, which is affiliated with the Tongji Medical College of Huazhong University of Science and Technology (Fan et al. 2023). Among 95 SCC samples, 6 HPV ( +) tumors were not detected HPV integration and 2 SCC were HPV negative (Fan et al. 2023).

### Analysis of integration sites

The breakpoints were annotated using the latest version of ANNOVAR in hg38 coordinates (Wang et al. 2010). Annotated genes with frequency ≥ 3 were defined as HPV integration hotspots. An integration site was confirmed to be adjacent to a genomic element when the element overlapped with the flanking region (200 bp) (Hu et al. 2015). Cytobands, transcription factor binding sites (TFBSs), repetitive regions, and CpG islands were downloaded from UCSC (http://hgdownload.cse.ucsc.edu/goldenPath/hg38/database). DNaseI hypersensitivity sites (DHSs), methylated or acetylated histone binding sites, and chromatin state of HeLa-S3 cells were downloaded from ENCODE (Bodelon et al. 2016). Functional annotation analysis of breakpoints was performed using R packages, and a heatmap was plotted using an online platform for data analysis and visualization (https://www.bioinformatics.com.cn). Protein–protein interaction network analysis (PPI) was constructed using the online tool STRING (https://www.string-db.org/), by setting medium confidence at 0.400 and excluding irrelevant genes (Zeng et al. 2021).

### Generation of random sites

Using R package, 1000 random sites within the human chromosome were generated, serving as a random control.

### Immunohistochemical (IHC)

To verify the impact of HPV integration on expression of most frequently integrated genes, we conducted IHC assay. The detailed process of IHC staining and analytical method was as described in this paper (TAN Yao et al. 2022). We chose five samples with these genes integrated as integrated group, and five samples with no same genes integrated as control group. We observed the tissue sections under microscope and selected five images per slide, and images were taken at 400 × magnification using the same light intensity and exposure time. Image J was used to analyze the integrated optical density (IOD) value of each image.

### Statistical analysis

SPSS 25.0 was used for statistical analysis. *P*-values were calculated using the *χ*^2^ test and corrected using Fisher’s exact test.

## Results

### Integration cytobands

**Fig. 1:**
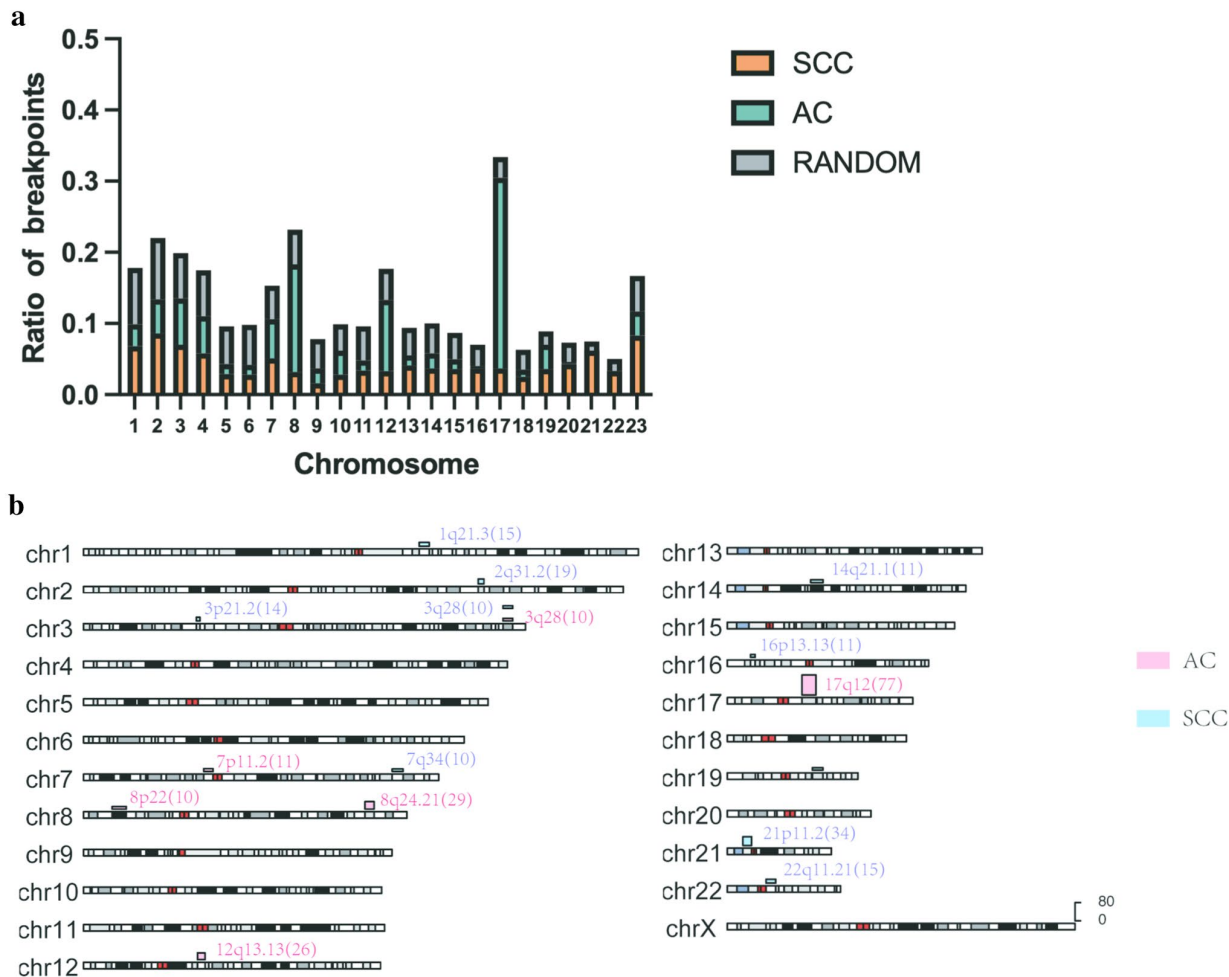
Distribution of breakpoints in human chromosomes. **a** Distribution of breakpoints in human chromosomes. The Y-axis represents the ratio of breakpoints, whereas the X-axis denotes different chromosomes. **b** Distribution of breakpoints in cytobands with frequency ≥ 10. The height of the histogram corresponds to the frequency of integration in the cytobands. Red histogram represents AC, whereas blue histogram represents SCC

Previous studies have shown that viral integration in cervical cancer frequently occurs in some specific cytobands, such as 8q24.21 and 3q28 (Bodelon et al. 2016). Therefore, we investigated cytobands with high number (frequency ≥ 10) of observed integration events in AC and SCC to understand the impact of HPV integration on carcinogenesis. In this study, we found integration events occur in all chromosomes (Fig. 1a). The results indicated that the integration points of AC tended to be located in chromosome 8, whereas in SCC, breakpoints tended to locate in chromosome 21. In AC, total 95 cytobands that not defined and 75 sub-bands were targeted, whereas in SCC 247 cytobands and 46 sub-bands were targeted. In AC, cytobands with a significant number of integration events (frequency ≥ 10) included 17q12 (26.5%, 77/291), 8q24.21 (10%, 29/291), 12q13.13 (9%, 26/291), 7p11.2 (3.8%, 11/291), 3q28 (3.4%, 10/291), 8p22 (3.4%, 10/291). In SCC, the most frequently integrated cytobands were 21p11.2 (4.5%, 34/760), 2q31.2 (2.5%, 19/760), 22q11.21 (2.0%, 15/760), 1q21.3 (2.0%, 15/760), 3p21.2 (1.8%, 14/760), 16p13.13 (1.4%, 11/760), 14q21.1 (1.4%, 11/760), 3q28 (1.3%, 10/760), 7q34 (1.3%, 10/760), 19q13.2 (1.3%, 10/760) (Fig. 1b). The percentage represented the ratio of breakpoints of all integration sites of all the cases. This revealed that HPV integration showed a different preference for specific cytoband in AC and SCC.

### HPV integration hotspots in AC and SCC

**Fig. 2:**
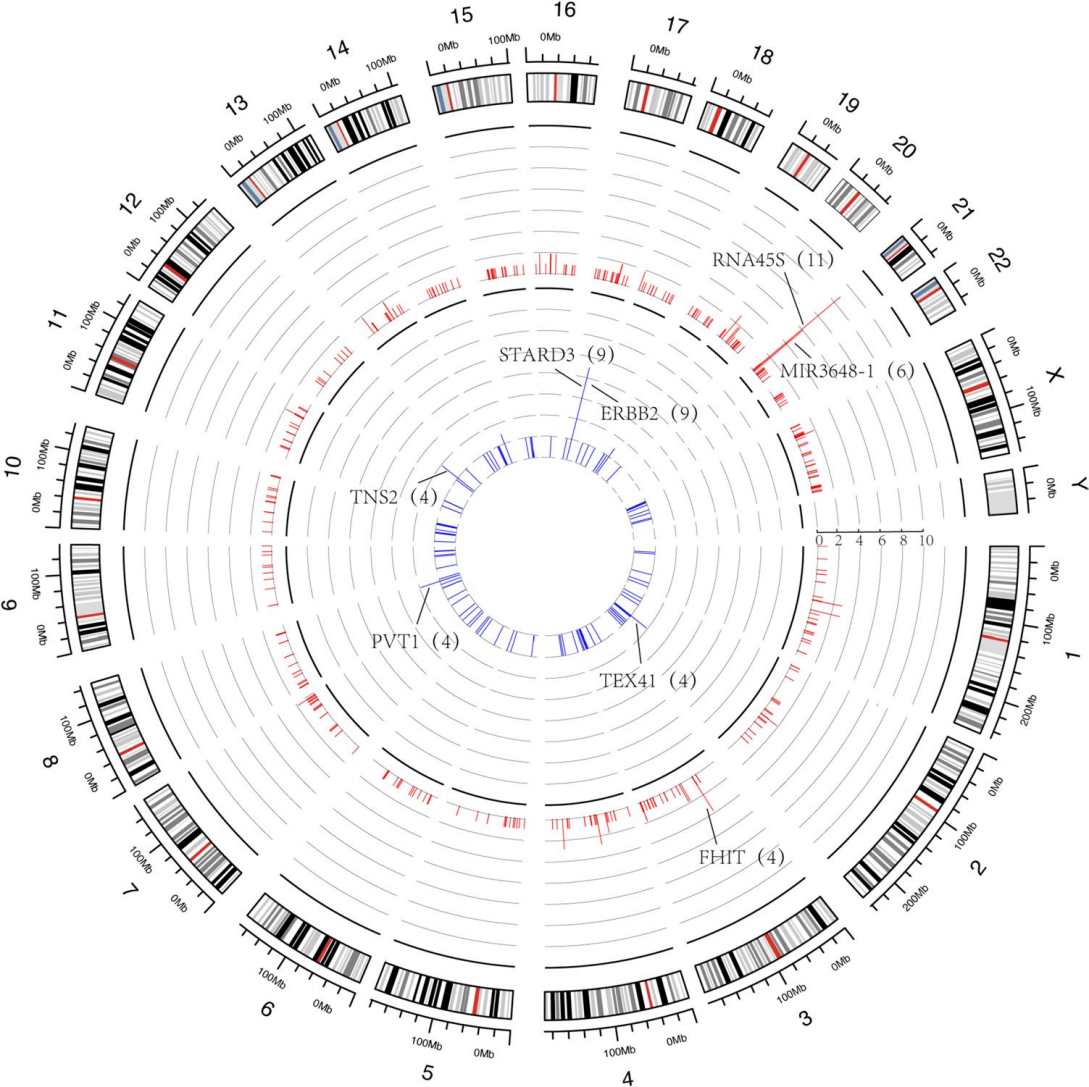
Frequency and distribution of integration breakpoints in the human genome. Each line represents the location of HPV integration in human chromosomes, and the length of the line represents the frequency of integration. In the inner circles, the red bars depict the frequency of SCC samples, whereas the blue bars depict the frequency of AC samples

Many studies agreed that HPV integration showed a strong tendency to be located in some hotspot genes (Hu et al. 2015; Bodelon et al. 2016). Identifying the different HPV integration hotspot genes in AC and SCC can help clarify the pathogenesis of cervical cancer. In AC, 12 hotspot genes (frequency ≥ 3) were detected. The most commonly integrated genes were STARD3 (17%, 9/53), ERBB2 (17%, 9/53), TNS2 (7.5%, 4/53), TEX41 (7.5%, 4/53), and PVT1 (7.5%, 4/53). In SCC, 10 hotspot genes were detected, including RNA45S (12.6%, 11/87), MIR3648-1 (6.9%, 6/87), and FHIT (4.6%, 4/87) (Fig. 2). We found that most hotspot genes were coding genes (8/12) in AC and noncoding genes (8/15) in SCC. The results indicated that HPV tended to integrate in different functional genes in AC and SCC.

### Distribution of breakpoints in exonic, intronic, and intergenic regions

**Fig. 3:**
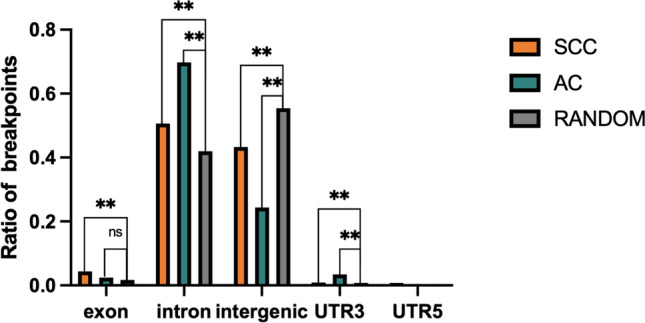
Distribution of integration sites in exonic, intronic, intergenic regions, 3’UTR and 5’UTR. The random integrated ratio of each region is counted according to the random distribution of breakpoints in the human genome (**P < 0.01)

We have explored the tendency of HPV integration for genes, but the locational relationship between integration sites and known human genes is unclear. In AC, we identified 291 valid integration sites, including 7 (2.4%) in exons, 203 (69.8%) in introns, 71 (24.4%) in intergenic regions, and 10 (3.4%) in UTR’3. In SCC, we identified 760 effective integration sites, including 33 (4.3%) in exons, 385 (50.7%) in introns, 329 (43.3%) in intergenic regions, 7 (0.9%) in UTR’3 and 6 (0.9%) in UTR’5. The results indicated that compared to the randomized control, both genomes of AC, and SCC showed a tendency to be integrated within genes, with integration sites in SCC more likely to be in exons (Fig. 3).

### Distribution of breakpoints in genomic elements

**Fig. 4:**
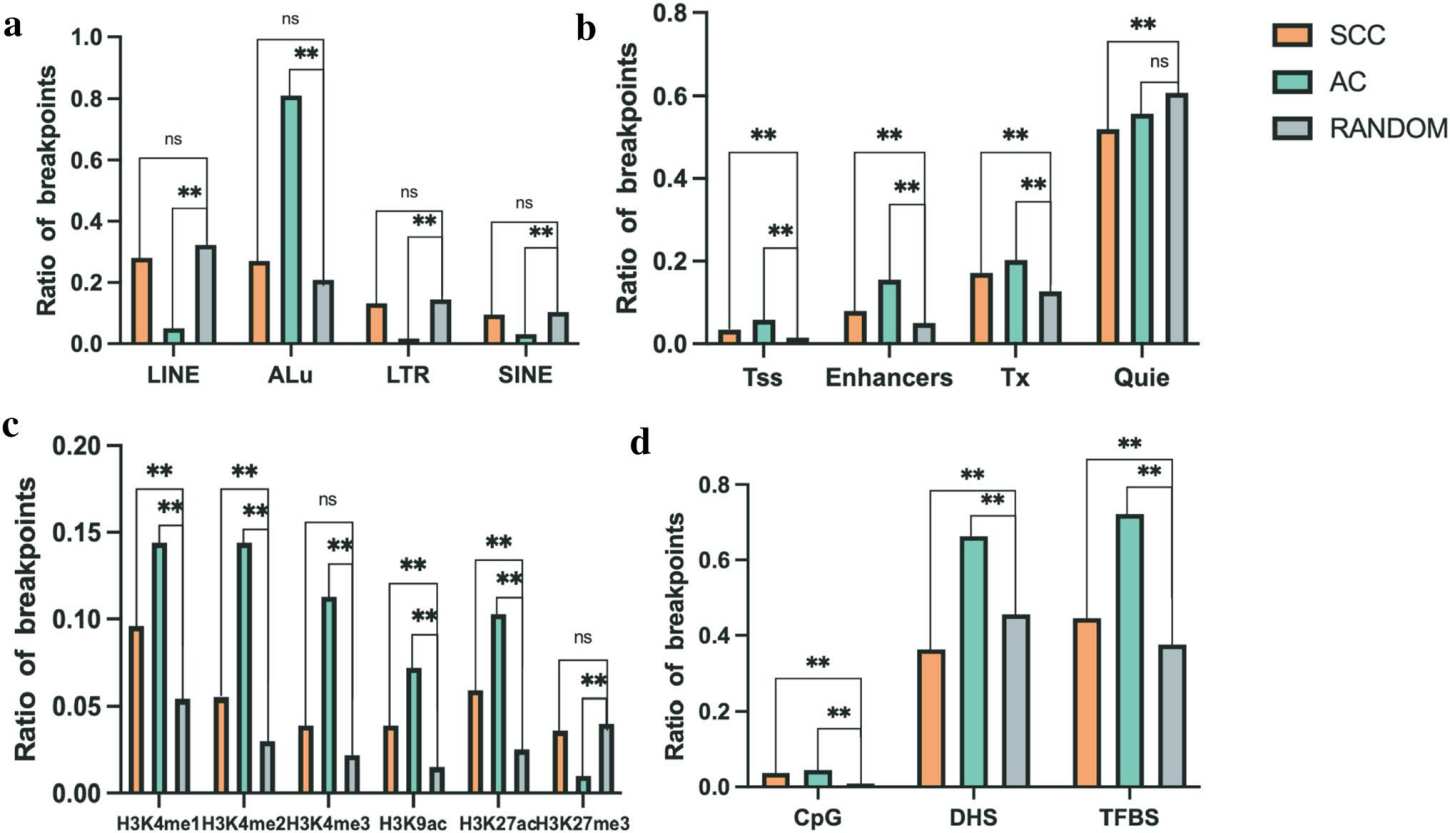
Distribution of integration sites in different genomic elements. The Y-axis represents the ratio of breakpoints, whereas the *X*-axis denotes different genomic elements. **a** Repetitive elements, **b** chromatin genome segmentation, **c** regions binding with histone markers, **d** and CpG enriched regions, DHS, and TFBS. (**P < 0.01)

Next, we examined the distribution of breakpoints for AC and SCC in several genomic features, including repetitive elements, chromatin genome segmentations, methylated or acetylated histone binding sites, CpG enriched regions, and DHSs. This helps understand common molecular features that may lead to HPV integration into the human genome (Bodelon et al. 2016) and cause the discrepancy between AC and SCC. ANNOVAR was used to perform region-based annotation (Wang et al. 2010). Repetitive elements are necessary for accurate genome replication and transmission to progeny cells (Shapiro and von Sternberg 2005). Owing to the same integration sites potentially annotated by the different region-element, we calculated the number of certain elements among the amount of all annotated elements. In this study, the integration sites in AC tended to locate in Alu element (80.9%,1184/1464). In AC, breakpoints occurred less frequently in LINE (Long interspersed nuclear elements; 5.1%, 75/1464), LTR (Long terminal repeat elements; 1.7%, 25/1464) and SINE (Short interspersed nuclear elements; 3.1%, 46/1464) compared to RANDOM (**P < 0.01, Fig. 4a). In SCC, HPV integration did not represent any intendency in repetitive regions.

Open chromatin regions, which are associated with transcriptional regulation, provide insights into genome function. Based on ENCODE chromatin genome segmentation information of HeLa-S3, the integration points of AC tended to located more in Enhancers (15.5%) than RANDOM (**P < 0.01). Compared to the randomized control, HPV tended to integrate in transcription start sites (Tss) flanking regions (frequency rate = 3.4% and 5.8% for SCC and AC, respectively) and Tx (frequency rate = 17.2% and 20.3% for SCC and AC, respectively) (Fig. 4b).

Histone modifications, particularly acetylation and methylation on specific amino acid residues, have been implicated in gene expression regulation (Bannister and Kouzarides 2011; Greer and Shi 2012). We analyzed the association between HPV integration and histone markers binding regions. The results showed that integration events in AC occurred more frequently than RANDOM in binding sites of H3K4me1 (14.4%), H3K4me2 (14.4%), H3K4me3 (11.3%), H3K9ac (7.2%) and H3K27ac (10.3%), but not H3K27me3 (1%) (Fig. 4c). Additionally, we examined other important genomic features, such as CpG enriched regions, TFBSs, and DHSs. CpG islands help identify promoters. TFBSs play a crucial role in regulating transcription (Bartlett et al. 2017). DHSs represent the susceptibility of chromatin (Lu and Richardson 2004). In our study, integration events in AC showed a preference for DHSs (66.3%) and TFBSs (72.2%). This indicated that in AC, HPV has a higher propensity for integration in regulatory regions compared to SCC.

**Fig. 5:**
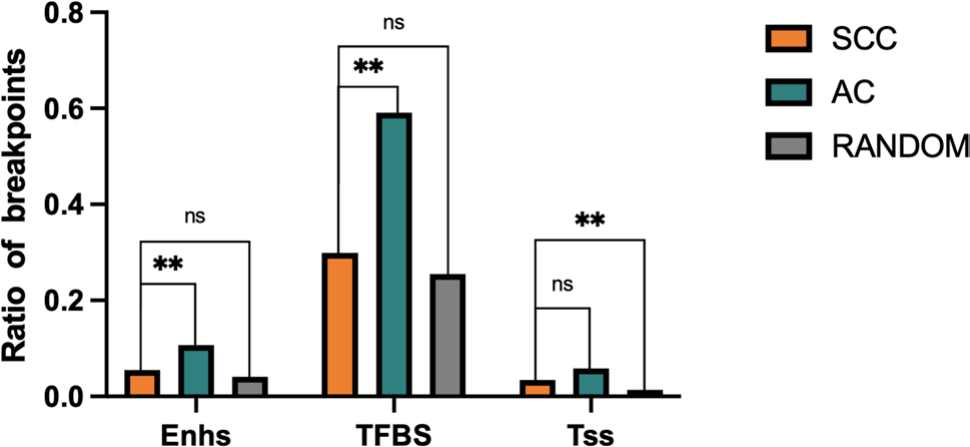
The relationship between these genomics elements and HPV integration sites. Breakpoints represent integration sites located both in DHSs and corresponding chromatin states. (**P < 0.01)

To analyze the relationship between these genomics elements and HPV integration sites with great confidence, we examined the co-occurrence of integration sites in DHSs and other genomic elements in AC and SCC (Fig. 5). We found that in AC, breakpoints more frequently co-occurred in DHSs and Enhancers (10.7%) or TFBS (59.1%) compared to SCC (**P < 0.01). This suggests a strong association between breakpoints in AC and these elements. Both AC and SCC breakpoints showed a tendency to co-occurrence in Tss (Frequency rate = 3.4% and 5.8% for SCC and AC, respectively), but the difference between AC and SCC was not statistically significant.

### Clinical characterization

A total of 66 AC samples were collected, of which 11 had invalid integration sites (frequency ≤ 3). HPV integration didn’t occur in two samples; therefore, 53 AC samples had valid breakpoints. A total of 95 SCC samples were collected, of which 87 were detected valid integration. 6 HPV ( +) tumors were not detected HPV integration and 2 SCC were HPV negative. The results revealed that in AC HPV integration had no significant difference with common clinical parameters, including age, differentiation, stage, HPV type and lymph metastasis (Table 1). In SCC, HPV integration only correlated with differentiation and the corresponding p-value was denoted by bold (*P* = 0.026).

**Table 1:** Relationship between clinicopathological parameters of AC and SCC, and HPV integration

Variable	Valid HPV integration of AC					Valid HPV integration of SCC				
	Negative		Positive		*P*-value	Negative		Positive		*P*-value
	No	%	No	%		No	%	No	%	
*Age*					0.115					0.522
< 40	8	32.0%	17	68.0%		1	7.7%	12	92.3%	
40–49	2	8.3%	22	91.7%		1	3.4%	28	96.6%	
> = 50	3	17.6%	14	82.4%		6	11.3%	47	88.7%	
*Differentiation*					0.144					**0.026**
Well	2	9.1%	20	90.9%		3	33.3%	6	66.7%	
Moderately or poorly	2	40%	3	60%		5	5.8%	81	94.2%	
*Stage*					1.000					0.273
CIS or stage 1	9	20%	36	20%		6	12.8%	41	87.2%	
Stage 2 +	1	80%	4	80%		2	4.8%	40	95.2%	
*Type of HPV*					0.881					1.000
HPV16	6	16.7%	30	83.3%		5	7.4%	63	92.6%	
HPV18	4	16.7%	20	83.3%		0	0.0%	6	100%	
Non-HPV16/18	1	25.0%	3	75.0%		1	5.3%	18	94.7%	
*Lymph metastasis*					0.171					0.676
Positive	1	100%	0	0%		1	4.5%	21	95.5%	
Negative	5	14.7%	29	85.3%		7	9.6%	66	90.4%	

### Breakpoints of HPV16 in SCC and AC

**Fig. 6:**
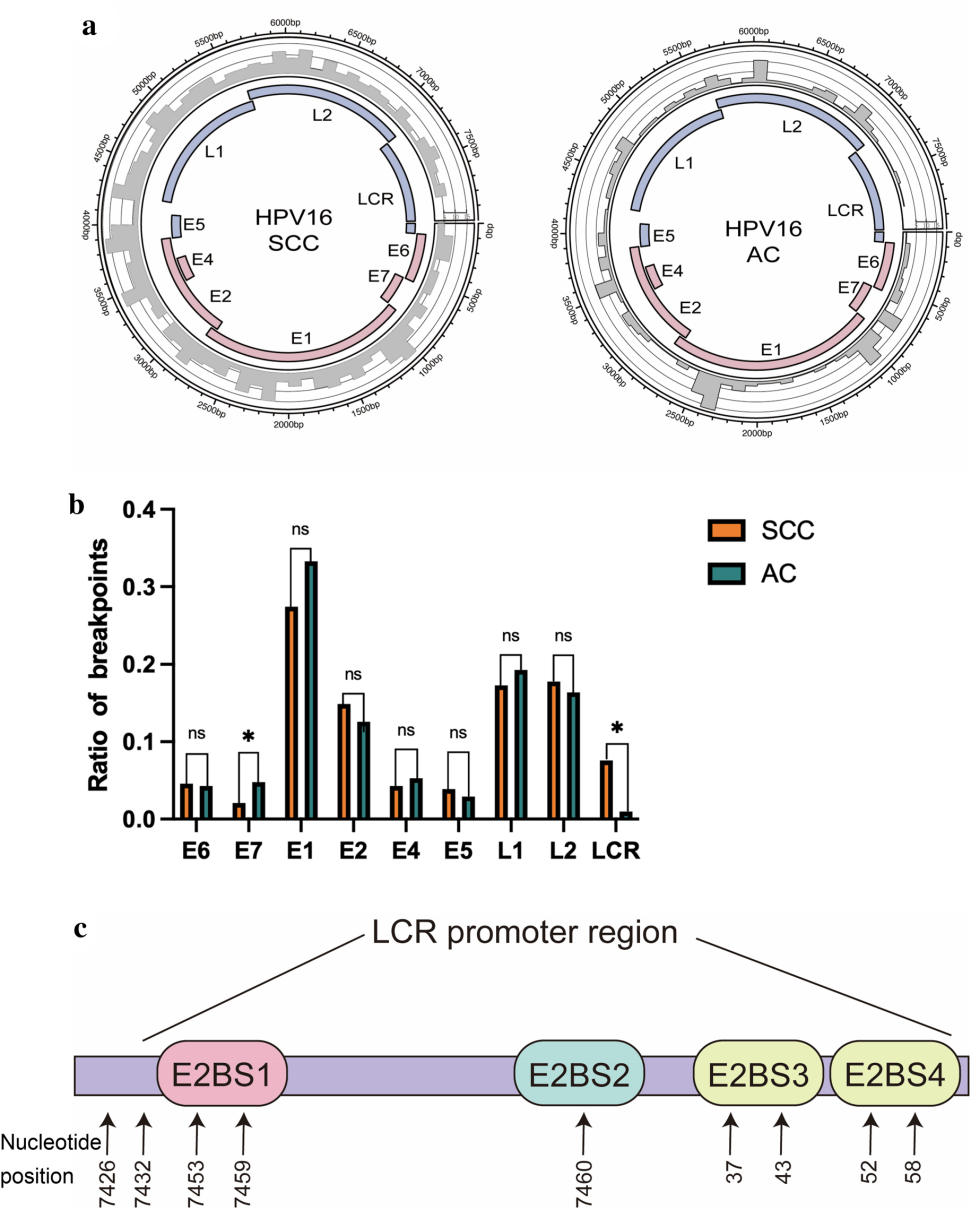
**a** Distribution of integration breakpoints in the HPV16 genome. Histograms (grey) of frequency of breakpoints in the samples are constructed for 100-bp intervals. Histogram axis units represent numbers of breakpoints, and outer DNA numbering is given in bases. HPV genes with different functions are colored. **b** Comparison of SCC and AC breakpoints ratio in the HPV16 genome. The Y-axis represents the ratio of HPV breakpoints, while the X-axis represents HPV16 genome. P-values are calculated by χ2test. **c** We represent the E2 binding sites in the HPV16 long control region (LCR). E2BS1 and promoter region covered from 7432 to 116 (Chaiwongkot et al. 2013)

On the other hand, we also studied the relationship between different genomic elements features of integration sites and HPV type. We found that there was no significant relation (supplementary Table S1). Then we analyzed the HPV breakpoints of AC and SCC. The results revealed that HPV16 breakpoints of AC intended to be located in E7 compared to that of SCC. Meanwhile, breakpoints of SCC were prone to be located in LCR compared to that of AC (Fig. 6a, b). Further we found that in SCC breakpoints of HPV16 tended to be located in EBS1 and promoter regions (nucleotide position: 7432–116) compared to AC (Fig. 6c). The percentage of breakpoints in this region among all breakpoints that were located in LCR in SCC and AC samples were respectively 78.4% (40/51) and 2.9% (1/34) (**P < 0.01). The breakpoints of HPV16 were shown in the supplementary material.

### Pathway analysis

**Fig. 7:**
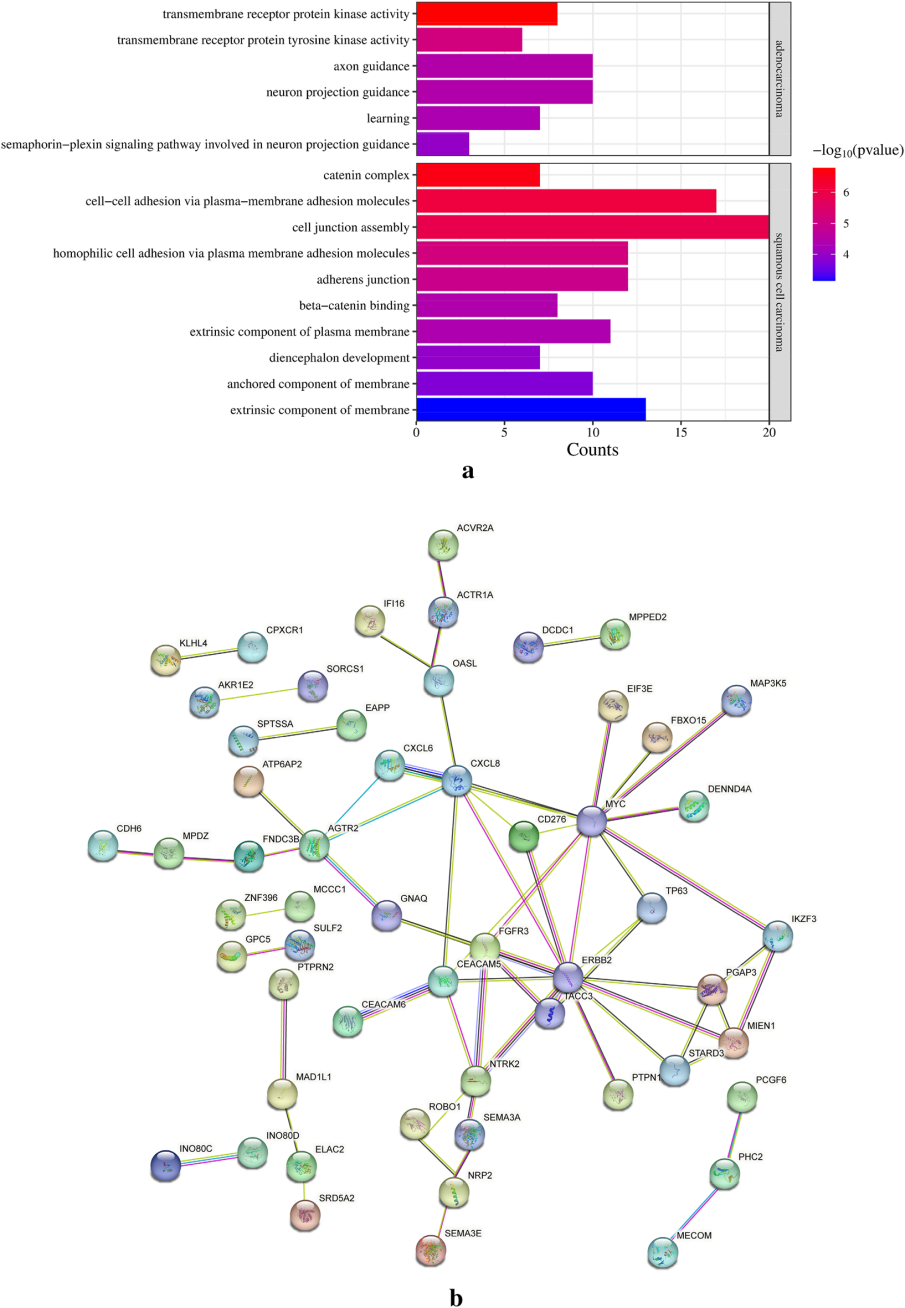
**a** GO enrichment pathway. The Y-axis represents enrichment pathways, whereas the X-axis represents counts of genes enriched for the corresponding GO terms. The color of the bars indicates *P*-values, with “red” indicating a significant enrichment of genes in the respective GO pathway. **b** PPI network for AC. The network was exported and visualized using online tool STRING. (https://www.string-db.org/)

To clarify the function of genes affected by HPV integration in AC and SCC, we used GO Pathway and PPI network analysis. GO analysis of hotspot genes revealed significant differences in enriched pathways between AC and SCC (Fig. 7a). Transmembrane receptor protein kinase pathway was enriched for AC; cell junction and cell–cell adhesion pathway were enriched for SCC. These results revealed that there was a significant difference between the enrichment pathway of AC and SCC. To understand the interaction of corresponding genes, the PPI network was constructed. Because we found no significant interaction between hotspot genes (frequency ≥ 3) of SCC, the picture is not shown here. 150 genes affected by HPV integration in AC samples were analyzed, and 115 genes were included in the network after the elimination of disconnected genes. The network is shown in Fig. 7b. The results indicated that in AC, genes ERBB2 and MYC were closely related to other affected genes by integration.

### Immunohistochemical (IHC) staining

**Fig. 8:**
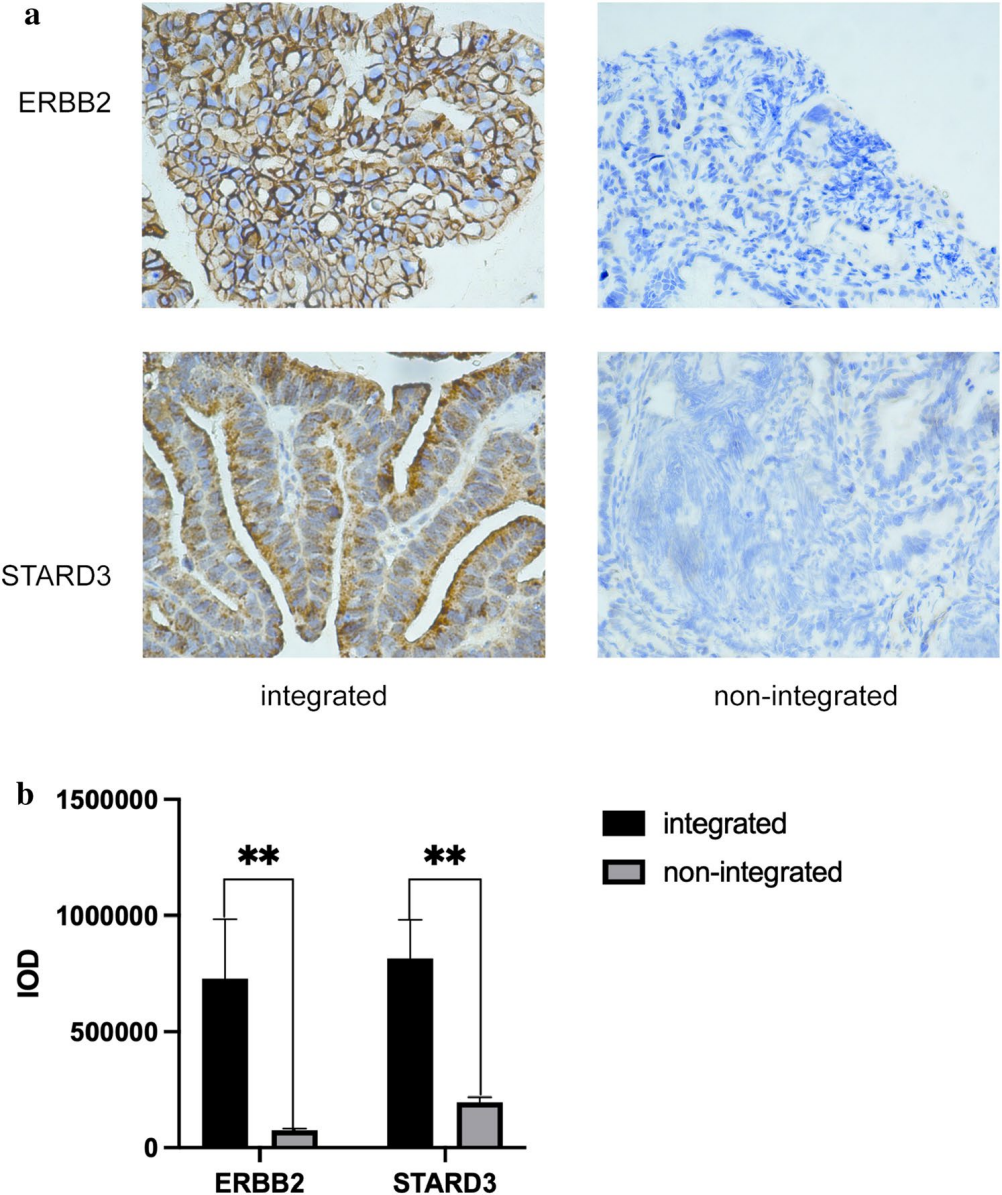
**a** From left to right, the IHC images of integrated group and negative group in sequence are represented. **b** The histogram represents the integrated optical density (IOD) of integrated group and non-integrated group. The bars in the picture represents the standard error (SE)

To preliminary verify the expression of most integrated genes in AC, we conducted IHC assay. The samples with ERBB2 integrated were the same as those with STARD3 integrated. ERBB2 is mainly expressed in cytomembrane and STARD3 is mainly existed in cytoplasm. Among five integrated samples, histocytes of one sample were most strongly stained with ERBB2 and STARD3 simultaneously compared to non-integrated samples (Fig. 8a). We used the integrated optical density (IOD) to quantize the expression of ERBB2 and STARD3. The average IOD of ERBB2, respectively, was 7.2 × 10^5^ and 7.6 × 10^6^ in integrated group and non-integrated group. The average IOD of STARD3, respectively, was 7.6 × 10^5^ and 2.1 × 10^5^ respectively. The results revealed that the expression of integrated genes was higher compared to non-integrated group (P < 0.01) (Fig. 8b).

## Discussion

To our knowledge, HPV is the main driver of cervical cancer and plays a significant role in the progression of cancers through various mechanisms. First, expression of the E6 and E7 oncogenes, that target p53 and Rb family proteins respectively, and lead to their degradation, results in uncontrolled proliferation (Vande Pol and Klingelhutz 2013; Roman and Munger 2013). In addition, HPV integration induces structural alterations and instability in the host genome (Porter and Marra 2022). These alterations may include gene amplification, deletion, extrachromosomal circular DNA formation, and focal rearrangement (Porter and Marra 2022). This study provides insights into HPV integration-mediated carcinogenesis in cervical cancer by analyzing the characteristics of HPV integration in SCC and AC from different genetic perspectives.

We examined the cytobands of HPV integration in AC and SCC samples. In AC, 17q12 (26.5%) was the most frequently integrated cytoband in AC in this study. Consistently, Hu supported the association between mutations in cytoband 17q12 and cervical cancer (Shi et al. 2013). Within cytoband 17q12, ERBB2 has been recognized as a potential target for treating cervical cancer (Oh et al. 2015). In this study, we found some sites were integrated frequently, such as chr17:39,695,542 (ERBB2) and chr17:39,647,045 (STARD3). Furthermore, we found that integration mainly occurs in intron of ERBB2, which is consistent with the results that integration sites tended to be located within introns. Martina Schmitz found that HPV integration sites were located within the intron sequences of known genes, and insertional mutagenesis could influence the function of genes (Schmitz et al. 2012).

In addition to 17q12 (26.5%), 8q24.21 (10%) was the second most frequently integrated bands. This result agrees with previous studies (Hu et al. 2015; Bodelon et al. 2016; Shi et al. 2013)_._ Among 28 integration sites on band 8q24.21, 8 were located within a distance of 2 kb to 460 kb upstream of the MYC gene and 20 were within a distance of 13 kb to 366 kb downstream of the MYC gene. These distances represent exceedingly short regions in genomic terms. M Peter had proposed that viral and MYC sequences were co-amplified in an amplicon between less than 50 and 800 kb in size (Peter et al. 2006). The amplification of the MYC oncogene was shown to significantly associate with heavily-rearranged extrachromosomal circular DNA (ecDNA) amplicons, which contained distal (> 1 Mb) amplified segments (Kim et al. 2020). So, we believed that the ecDNA amplicon could simultaneously promote viral oncogene and MYC gene co-amplifying. This may play an important role in carcinogenesis of HPV.

21p11.2 (4.5%) was the most frequently integrated cytoband in SCC. In this cytoband, genes encoding 45S ribosomal RNA (RNA45S rDNA), which harbor strong promoters, are abundant. Viral sequences could utilize hosts’ promoters to express its proteins. Inagaki proposed that two distinct cDNA clones of the HPV type 18 transcript are present in HeLa cells (Inagaki et al. 1988). These clones contain the viral genes E6 and E7, as well as a shared human sequence located at chr8:127,228,560 -127,229,294 (Inagaki et al. 1988). They proposed that HPV 18 integrates into the lncRNA and utilizes its promoter to express the viral oncoprotein E6 and E7 in HeLa cells (Inagaki et al. 1988). In our study, viral sequence integrated in 21p11.2 cytoband could likewise utilize RNA45S rDNA’ promoters to express viral proteins in SCC.

Another cytoband 3q28 in SCC has been reported in previous study (Bodelon et al. 2016). In 3q28, mutations in TP63, a member of the p53 family, are associated with various cancers: notably, lung and bladder cancers (Lu et al. 2018; Wang et al. 2016). Min Liu hypothesized that highly expressed HPV-human fusion transcripts could promote overexpression of E6*I and E7 and inhibit the transcription of tumor suppressor genes TP63 and P3H2 (Liu et al. 2023). In our study, 3 breakpoints were located in intergenic regions between TP63 and P3H2. Therefore, this indicated that HPV-human fusion transcripts may play an important role in carcinogenesis of SCC, by promoting overexpression of viral oncoproteins and inhibiting the transcription of tumor suppressor genes. In conclusion, we proposed that HPV integration in different cytobands promotes the progression of cancers through different mechanisms and shows a tendency for integration into specific cytobands.

We identified integration sites within regions with various elements, such as repetitive elements, open chromatin (DHSs), and chromatin genome segmentation (chromatin 18-state of HeLa-S3). The findings revealed that breakpoints in AC tended to locate in Alu elements, Enhancers, DHSs, TFBSs and H3K4 methylated histone binding sites compared with that in SCC. Among these elements, DHSs serve as a global measure of chromatin accessibility (Dao et al. 2021), providing insights into the functional elements within the genome. We identified Enhs, TFBS, and Tss with great confidence by intersecting DHSs with chromatin state regions identified in the ENCODE project (Fig. 5). This suggested that in AC, HPV has a higher propensity for integration in regulatory regions, potentially influencing host gene expression and cellular processes.

HPV integration at recurrent loci may provide selection advantage to host cells (Schmitz et al. 2012), leading to the recurrence of hotspot genes. We found that hotspots were coding genes (8/12) in AC and noncoding genes (8/15) in SCC. In AC samples, ERBB2 (9) and STARD3 (9) were the most integrated genes. The mutation rate of ERBB2 was reported to be significantly higher in AC than SCC (Xiang et al. 2018). Moreover, the positive rate of ERBB2 protein in AC was more than one-half (Shi et al. 2021). Further through GO analysis, we found that integrated genes in AC enriched in transmembrane receptor protein kinase pathway, and that of SCC enriched in cell junction and cell–cell adhesion pathway. Through the PPI network for AC, we found ERBB2 was closely related to other genes affected by HPV integration. STARD3 (9) is a member of the lipid transporter subfamily and plays a critical role in the synthesis and maintenance of cholesterol balance (Wilhelm et al. 2017). Studies on STARD3 have focused on gastric carcinoma and breast cancer (Wilhelm et al. 2017; Lodi et al. 2023); however, studies on cervical cancer are lacking. Previous studies have shown that STARD3 co-amplified and co-expressed with HER2 in breast cancer (Bieche et al. 1996; Tomasetto et al. 1995). The reduction of STARD3 expression in HER2-positive cancer cell lines inhibit their growth (Kao and Pollack 2006; Sahlberg et al. 2013). In this study, STARD3 was related to ERBB2 closely in the PPI network. Through IHC assay, we found that ERBB2 and STARD3 were most strongly expressed in the same sample compared to no integrated samples. And the rest integrated samples have also difference with non-integrated group (P < 0.01). We further analyzed the integrated sites. All samples with ERBB2 integrated have the same breakpoints in chr17: 39,695,542/39695556/39703981 (ERBB2) and chr17: 39,647,045 (STARD3). The stained most strongly sample not only have these sites, but also other different sites. This sample was also detected mostly integrated sites of STARD3 and the highest expression of STARD3. It was not clear why the expression of integrated genes was mostly increased in the same sample. The amounts of integrated sites or certain specific site maybe reasons and more researches need to be done. In a word, the samples with integration in ERBB2 was same as that with integration in STARD3 and they both overexpressed. We strongly believed that STARD3 co-amplified and co-expressed with ERBB2 in AC. Meanwhile, the FIGO stage of the most strongly stained sample was more advanced. Of particular note is that owing to small set of samples, more researches need to be done. STARD3 and ERBB2 potentially were recognized as novel candidate biomarkers of AC. RAD51B (3) is known as a hotspot gene (Li et al. 2018). It plays a critical role in homologous recombinational repair of DNA double-strand breaks (important for genomic stability and a hallmark of cancer) (Hang et al. 2016). In this study, HPV integration sites were found in intron 7 of RAD51B-204 (the main transcript) and its breakage may be promoting progression of cancer. Therefore, we hypothesized that HPV integration may lead to the dysregulation of oncogenes, that plays a more important role in HPV-related AC.

RNA45S rDNA (11) serves as the most frequently integrated hotspot gene of SCC. One-half of the integration sites were located in intergenic regions between RNA45S (distance = minimum 2 kb and maximum 400 kb in chr21) and MIR3648-1 (distance = minimum 510 kb and maximum 530 kb in chr21). rDNA is located in fragile sites in the chromosomes (Sidler 2016), making it susceptible to breakage and HPV integration. Genes encoding 45S ribosomal RNA are abundant in eukaryotic genomes (Havlova and Fajkus 2020). The change of rDNA expression due to integration maybe negligible, but as mentioned above, rDNA possesses powerful promoters that can promote the overexpression of viral oncogenes. In SCC, viral oncogenes may utilize the powerful hosts’ promoters to express viral oncogenes and the overexpression of viral oncogenes may play a more important role in the development of carcinogenesis in SCC than that of AC.

We studied the relationship between clinicopathological parameters and HPV integration and found that HPV integration correlated with FIGO stage and pathology (**P < 0.01). However, the amount of AC samples was a little and some data of AC were missing, this result need more study to verify. Then, we investigated the association between the breakpoints of HPV16, AC and SCC. The results revealed that HPV16 breakpoints of AC intended to locate in E7 compared to that of SCC. Meanwhile, breakpoints of SCC prone to occur in LCR compared to that of AC (Fig. 6a, b). Previous study has revealed that E2 bond with high-affinity E2BS1 and the p97 promoter was activated (Reuschenbach et al. 2015). Then the level of E2 enhanced. E2 then also bonded to the low-affinity E2BS3 and E2BS4, and this resulted in inhibition of the p97 promoter (Reuschenbach et al. 2015). Arkom Chaiwongkot proved that E2BS1 and promoter region covered from 7,432 to 116 (Chaiwongkot et al. 2013). We found breakpoints of HPV16 in SCC tended to locate in the E2BS1 and promoter region, which destroyed the binding of E2 and E2BS1. The activation of p97 promoter that promoted the expression of E6/7 was unable. Therefore, this result was corresponding to our hypothesis that viral oncogenes may utilize the powerful hosts’ promoters to express viral oncogenes.

In our study, we focused on the characterization of HPV integration sites in AC and SCC. Notably, we only included cancer samples and did not enroll non-cancer samples with HPV integration. Thus, the characteristics of the observed regions probably contribute to carcinogenesis, instead of conferring susceptibility to HPV integration. We analyzed the results and speculated the carcinogenic mechanisms of HPV integration in SCC and AC focusing on highly frequent integration cytobands, characterization of genomic elements, integration of hotspot genes, enrichment pathways, and clinicopathological parameters. Meanwhile, we analyzed the HPV16 breakpoints in AC and SCC. In conclusion, the HPV-mediated carcinogenic mechanisms in SCC and AC were different. We proposed that the overexpression of viral oncogenes plays a more important role and it may utilize the powerful hosts’ promoters to express viral oncogenes in carcinogenesis in SCC compared to AC. In AC, HPV integration may affect hosts’ oncogenes, such as ERBB2 and STARD3. And the dysregulation of oncogenes may primarily contribute to progression of AC.

## Supplementary Information

Below is the link to the electronic supplementary material.

Supplementary file1 (PDF 82 KB)

Supplementary file2 (XLSX 47 KB)

## Data Availability

The datasets generated and analyzed during the current study are available in the Supplementary Information.
