# Supplementary material for: Characteristics of HPV integration in cervical adenocarcinoma and squamous carcinoma
Source: J Cancer Res Clin Oncol. 2023 Nov 15;149(20):17973–86. doi: 10.1007/s00432-023-05494-4 (PMC10725361; doi:10.1007/s00432-023-05494-4)
Supplement: Supplementary file 1 — Supplementary file1 (PDF 82 KB) [file 432_2023_5494_MOESM1_ESM.pdf]

# Characteristics of HPV Integration in Cervical Adenocarcinoma and Squamous Carcinoma

**Yuxin Bi [1 2], Junbo Hu [4], Ling Zeng [1 5], Gang Chen [3], Hongning Cai [1 2], Huang Cao [1 2 5], Quanfu Ma [1 2], Xufeng Wu [1 2]**

1 Maternal and Child Health Hospital of Hubei Province, Huazhong University of Science and Technology, Wuhan, China;
2 Hubei Clinical Medical Research Center for Gynecologic Malignancy, Wuhan, China;
3 Department of Obstetrics and Gynecology, Tongji Hospital, Tongji Medical College, Huazhong University of Science and Technology, Wuhan, Hubei, China;
4 Department of Pathology, Maternal and Child Health Hospital of Hubei Province, Huazhong University of Science and Technology, Wuhan, China;
5 Medical Genetics Center of Hubei Province, Wuhan, China;

**Supplementary Table S1** Relationship between genomic elements features of integration sites and HPV type.

| | | AC | | | | | | SCC | | | | | |
|---|---|---|---|---|---|---|---|---|---|---|---|---|---|
| | | | Negative | | Positive | | | | Negative | | Positive | | |
| | HPV type | total | num | % | num | % | Pvalue | total | num | % | num | % | Pvalue |
| | HPV16 | 96 | 24 | 25 | 72 | 75 | | 571 | 292 | 51.1 | 279 | 48.9 | |
| DHS | HPV18 | 64 | 25 | 39.1 | 39 | 60.9 | 0.161 | 21 | 9 | 42.9 | 12 | 57.1 | 0.667 |
| | others | 9 | 3 | 33.3 | 6 | 66.7 | | 93 | 50 | 53.8 | 43 | 46.2 | |
| | HPV16 | 96 | 79 | 82.3 | 17 | 17.7 | | 571 | 526 | 92.1 | 45 | 7.9 | |
| Enhs | HPV18 | 64 | 56 | 87.5 | 8 | 12.5 | 0.722 | 21 | 18 | 85.7 | 3 | 14.3 | 0.446 |
| | others | 9 | 8 | 88.9 | 1 | 11.1 | | 93 | 87 | 93.5 | 6 | 6.5 | |
| | HPV16 | 96 | 32 | 33.3 | 64 | 66.7 | | 571 | 313 | 54.8 | 258[a] | 45.2 | |
| TFBS | HPV18 | 64 | 30 | 46.9 | 34 | 53.1 | 0.204 | 21 | 7 | 33.3 | 14[a] | 66.7 | **0.002** |
| | others | 9 | 4 | 44.4 | 5 | 55.6 | | 93 | 65 | 69.9 | 28[b] | 30.1 | |
| | HPV16 | 96 | 91 | 94.8 | 5 | 5.2 | | 571 | 547 | 95.8 | 24 | 4.2 | |
| Tss | HPV18 | 64 | 62 | 96.9 | 2 | 3.1 | 0.443 | 21 | 21 | 100 | 0 | 0 | 0.087 |
| | others | 9 | 8 | 88.9 | 1 | 11.1 | | 93 | 93 | 100 | 0 | 0 | |
| | HPV16 | 96 | 78 | 81.3 | 18 | 18.8 | | 571 | 512 | 89.7 | 59 | 10.3 | |
| H3K4me1 | HPV18 | 64 | 55 | 37.9 | 9 | 14.1 | 0.27 | 21 | 17 | 81 | 4 | 19 | 0.165 |
| | others | 9 | 6 | 66.7 | 3 | 33.3 | | 93 | 87 | 93.5 | 6 | 6.5 | |
| | HPV16 | 96 | 83 | 86.5 | 13 | 13.5 | | 571 | 534 | 93.5 | 37 | 6.5 | |
| H3K4me2 | HPV18 | 64 | 59 | 92.2 | 5 | 7.8 | 0.499 | 21 | 20 | 95.2 | 1 | 4.8 | 0.268 |
| | others | 9 | 8 | 88.9 | 1 | 11.1 | | 93 | 91 | 97.8 | 2 | 2.2 | |
| | HPV16 | 96 | 88 | 91.7 | 8 | 8.3 | | 571 | 544 | 95.3 | 27 | 4.7 | |
| H3K4me3 | HPV18 | 64 | 61 | 95.3 | 3 | 4.7 | 0.434 | 21 | 21 | 100 | 0 | 0 | 0.21 |
| | others | 9 | 8 | 88.9 | 1 | 11.1 | | 93 | 92 | 98.9 | 1 | 1.1 | |
